# Neurobehavioral Outcomes Relate to Activation Ratio in Female Carriers of Fragile X Syndrome Full Mutation: Two Pediatric Case Studies

**DOI:** 10.3390/ijms26020771

**Published:** 2025-01-17

**Authors:** Elisa Di Giorgio, Silvia Benavides-Varela, Annamaria Porru, Sara Caviola, Marco Lunghi, Paola Rigo, Giovanna Mioni, Giulia Calignano, Martina Annunziata, Eloisa Valenza, Valentina Liani, Federica Beghetti, Fabiola Spolaor, Elisa Bettella, Roberta Polli, Zimi Sawacha, Alessandra Murgia

**Affiliations:** 1 Department of Developmental Psychology and Socialisation, University of Padova, 35131 Padova, Italy; silvia.benavidesvarela@unipd.it (S.B.-V.); annamaria.porru@unipd.it (A.P.); sara.caviola@unipd.it (S.C.); marco.lunghi@unipd.it (M.L.); paola.rigo.1@unipd.it (P.R.); giulia.calignano@unipd.it (G.C.); martina.annunziata@studenti.unipd.it (M.A.); eloisa.valenza@unipd.it (E.V.); 2 Padova Neuroscience Center, University of Padova, 35131 Padova, Italy; 3 Department of Women’s and Children’s Health, University of Padova, 35128 Padova, Italy; valentina.liani@aopd.veneto.it (V.L.); fabiola.spolaor@unipd.it (F.S.); alessandra.murgia@unipd.it (A.M.); 4 Department of General Psychology, University of Padova, 35131 Padova, Italy; giovanna.mioni@unipd.it; 5 Department of Information Engineering, University of Padova, 35131 Padova, Italy; federica.beghetti@studenti.unipd.it (F.B.); zimi.sawacha@unipd.it (Z.S.); 6 Neurodevelopment Molecular Genetics Laboratory, Department of Women’s and Children’s Health, University of Padova, 35127 Padova, Italy; elisa.bettella@unipd.it (E.B.); roberta.polli@unipd.it (R.P.); 7 Istituto di Ricerca Pediatrica, Foundation-Città della Speranza, 35127 Padova, Italy

**Keywords:** full mutation, activation ratio, neurobehavioral outcome, multi-method research protocol, Fragile X syndrome

## Abstract

Fragile X syndrome (FXS) is a genetic neurodevelopmental disorder that causes a range of developmental problems including cognitive and behavioral impairment and learning disabilities. FXS is caused by full mutations (FM) of the *FMR1* gene expansions to over 200 repeats, with hypermethylation of the cytosine–guanine–guanine (CGG) tandem repeated region in its promoter, resulting in transcriptional silencing and loss of gene function. Female carriers of FM are typically less impaired than males. The Activation Ratio (AR), the fraction of the normal allele carried on the active X chromosome, is thought to play a crucial modifying role in defining phenotype severity. Here, we compare the cognitive, neuropsychological, adaptive, and behavioral profile of two FXS girls (10 and 11 years old) with seemingly identical *FMR1* genotypic profile of FM but distinctive AR levels (70% vs. 30%). A multi-method protocol, combining molecular pathophysiology and phenotypical measures, parent reports, lab-based tasks, gait analyses, and eye-tracking was employed. Results showed that lower AR corresponds to worse performances in most (cognitive, neuropsychological, adaptive, behavioral, social, mathematical skills), but not all the considered areas (i.e., time perception and gait analysis). These observations underscore the importance of AR as a phenotypic modifying parameter in females affected with FXS.

## 1. Introduction

Fragile X Syndrome (FXS) is a genetic neurodevelopmental disorder (NDD) that causes a range of development problems, which may include cognitive impairment and intellectual disability (ID), learning disabilities, and behavioral disorders (i.e., social anxiety, Attention Deficit/Hyperactivity Disorder (ADHD), and autism spectrum disorder (ASD)) [1]. FXS is caused by a pathological expansion of a Cytosine–guanine–guanine (CGG) trinucleotide repeated in the promoter region of the *FMR1* gene. The repeat is classified into four groups based on the size of the repeat: normal alleles (5–44 repeats), intermediate alleles (45–54 repeats), premutation alleles (55–200 repeats), and full mutant alleles (>200 repeats). Over 200 CGG repeat expansions result in a Full Mutation (FM, estimated incidence from 1:4000 to 1:7000 in males and from 1:6000 to 1:11,000 in females). These expansions result in extensive methylation of the *FMR1* gene, with subsequent transcriptional silencing and lack or reduction in the Fragile X Messenger RibonucleoProtein 1 (FMRP) production; this is an mRNA-binding protein, which plays a key regulatory role in synaptic function [2].

The cognitive and behavioral phenotype associated with FM includes delays in motor and language milestones achievement, sensory deficits, mild to severe ID, as well as severe behavioral alterations, such as social anxiety, Attention Deficit/Hyperactivity Disorder (ADHD), and ASD [3]. There is a considerable variation between females and males, with female carriers generally less affected than males. Specifically, although about half of females with FXS are diagnosed with ID, the female phenotype is more frequently associated with learning disabilities, behavioral and socio-emotional problems, difficulties establishing social interactions, as well as mental health issues, such as anxiety and depression [4].

Whilst isolation of the *FMR1* gene has led to a better understanding of the molecular basis for phenotypic variability, other molecular variables (beyond CGG repeats and methylation status) may also impact cognitive and behavioral profiles. The phenotypes’ variation among females with full mutation FXS can, at least in part, be attributed to a phenomenon known as X inactivation [5,6]. The activation ratio (AR), which represents the fraction of the normal allele carried on the active X chromosome, has been shown to be a relevant clinical parameter in many disorders involving X-linked genes [7,8,9,10,11,12]. In the case of the *FMR1* gene, if skewed X inactivation (XCI) occurs, resulting in more of the normal allele on the inactive X, the lack of FMRP in FM allele carriers may result in a more severe clinical phenotype. Despite its relevance, AR data are often missing from the molecular reports which results in a relative paucity of information on this front. Even though few studies have investigated AR in females with premutation [9], the role of AR in modeling the cognitive and behavioral phenotype in FM FXS females is still controversial.

A study conducted by Taylor et al. [13] did not find a significant correlation between intellectual quotient level (IQ) and AR in FM females. On the other hand, other studies found a significant association between AR and the overall IQ [10,14]. Reiss et al. [14] showed that AR (but not repeat size) was correlated with IQ in girls with FM, suggesting that such a parameter would be a stronger predictor of those measures in which FM females show the greatest impairment (i.e., visuo-spatial, and mathematical skills) as opposed to those skills that appear to remain relatively intact (i.e., vocabulary). Likewise, a positive correlation between AR and executive functions was observed in FM females [12]. Differently, Cornish et al. [11] found no evidence of an association between AR and performance on a range of standardized visuo-spatial tasks, suggesting that AR serves as a predictor of general intellectual functioning rather than specific cognitive deficits in FM females. In addition, reported case studies support the hypothesis that the different phenotypes in female carriers of FM are primarily caused by unequal AR. For example, one case study described two sisters with full mutations of *FMR1* with different phenotypes. One sister, with complete inactivation of the normal X chromosome, had severe ID and phenotypic features like those observed in FXS males. On the contrary, her sister, with the normal X chromosome active in 70% of her cells, showed normal IQ and learning disabilities [15,16,17].

Taken together, the literature suggests that despite the importance of AR in characterizing the functional level of FM females, its effect on the phenotype is not well understood. Therefore, the present study aims to investigate the impact of AR on cognitive, neuropsychological, adaptive, and behavioral profiles by comparing two FXS girls (10 and 11 years old) with seemingly identical *FMR1* genotypic profiles of FM in the presence of opposite AR levels (70% vs. 30%). A multidisciplinary and multi-method research protocol was used, combining molecular pathophysiology and phenotypic measures, parent reports, lab-based experimental tasks, gait analyses, and eye-tracking.

The data presented in this paper are part of a larger ongoing project that aims at characterizing the genotype–phenotype relationship in a pediatric population with FXS (males and females with both premutation and FM) regularly followed up at the Fragile X Centre of Padua (Procedures and Methods for all the areas under investigation are described in SI). Inclusion criteria for participants were as follows: (1) having a diagnosis of a *FMR1* mutation (premutation and full mutation with or without a mosaicism condition), (2) being between 3 and 17 years of age, and (3) having a mental age > 5 years as measured by standardized IQ and adaptive skills tests. Exclusion criteria included the following: (1) being diagnosed with other genetic conditions, (2) having a mental age < 5 years, (3) having other comorbidities that precluded testing (major motor, visual, and hearing difficulties). Specifically, the two cases reported in the present study were selected because they both (1) met the inclusion criteria of the general project, (2) were of comparable chronological age, and, more importantly, (3) carried the FM, with an opposite AR (30% vs. 70%).

Appropriate informed consent was obtained for Proband 1 and Proband 2 who were enrolled in the study for participation, scientific use of the data, and publication.

## 2. Description of the Two Cases

Proband 1 and Proband 2 were evaluated during a routine clinical setting at the Department of Women’s and Children’s Health and at the Department of Developmental Psychology and Socialization (University of Padova). The cognitive and behavioral profiles of the two girls are very different.

Proband 1 is physically a well-developed 10.1-year-old girl (age at diagnosis, 4 y) with mild hypotonia and no major anomalies. She is a second-born child of healthy, unrelated parents and has a 13-year-old brother with an FM condition (fully methylated). Born at term (39 Gestational Weeks) by cesarean section, with a birth weight of 3200 g. Prenatal and perinatal history were not remarkable. No congenital defects were found. Her general and neurological examinations were in range. Proband 1 acquired the psychomotor stages of development late, with difficulties in the motor, language, and social areas. With age, emotional dysfunctions, such as an inadequate response to a situation, attention and concentration difficulties, anxiety, and mild social withdrawal, as well as language delay, became apparent. At the time of examination, she displays moderate to severe social avoidance, compatible with social anxiety. Since the age of 12 months, she benefited from speech therapy and psychomotricity. Family history did not reveal any pathologies of neurological or psychiatric interest, except for maternal uncle with referred learning impairments. The proband’s mother (49y) is a PM carrier with Fragile X Associated Primary Ovarian Insufficiency (FXPOI). She is healthy and has a normal intelligence.

On the contrary, Proband 2 is an 11.8-year-old typically developing girl (age at diagnosis, 7 y). She is a first-born child of healthy, unrelated parents and has an 8-year-old brother with an FM condition (fully methylated). Born at term (41 Gestational Weeks) by cesarean section, with a birth weight of 3600 g. Prenatal and perinatal history were not remarkable. No congenital defects were found. Her general and neurological examinations were in range. There are no dysmorphisms or clinical comorbidities. She had a typical psychomotor and social development, and, therefore, did not benefit from any rehabilitation treatment. However, low self-esteem and performance anxiety are reported. Family history did not reveal any pathologies of neurological or psychiatric interest. The proband’s mother (44 y) is a PM carrier. She is healthy and has a normal intelligence.

### 2.1. Molecular Investigation

Molecular testing for Fragile X Syndrome (FXS) was performed using the AmplideX^®^ PCR assay (Asuragen, Austin, TX, USA) for CGG repeat sizing, and the *FMR1* promoter methylation status was evaluated with the AmplideX^®^ mPCR *FMR1* Kit (Asuragen^®^, Austin, TX, USA), according to the manufacturer’s protocol (see SI).

- Proband 1

The molecular assays applied to Proband 1 revealed two distinct alleles in the *FMR1* gene. The first allele contained 29 CGG repeats, characteristic of a normal range, while the second exhibited a repeat expansion greater than 200 repeats, accompanied by promoter hypermethylation, causing transcriptional silencing. The X chromosome inactivation (XCI) analysis demonstrated non-random inactivation, with an AR of 30%, suggesting predominant inactivation of the X chromosome carrying the normal allele.

- Proband 2

In Proband 2, the analysis identified two alleles in the *FMR1* gene: a typical allele with 29 CGG repeats and a second allele with an expansion exceeding 200 repeats, which was hypermethylated at the promoter region. This epigenetic alteration resulted in transcriptional silencing of *FMR1*. The X chromosome inactivation (XCI) analysis showed non-random inactivation, with an AR of 70%, indicating a preference for inactivating one X chromosome, potentially leading to higher functional expression of the gene and affecting the clinical phenotype.

### 2.2. Cognitive, Neuropsychological, Adaptive and Behavioral Profile

Cognitive, neuropsychological, behavioral, and adaptive profiles have been assessed by means of standardized tests and parent-report questionnaires. Specifically, (i) the Wechsler Intelligence Scales for Children for IQ [18]; (ii) Child Behavior Checklist (CBCL) [19], Conners’ parent rating scale-revised (CPRS-R:L) for the behavioral profile [20], and (iii) Vineland Adaptive Behavioral Scales (VABS-2) for adaptive profile [21]. Clinical data and scores of IQ, some neuropsychological functions (i.e., visual and auditory attention, visuo-spatial and verbal memory), behavioral and adaptive functioning are reported in Table 1, Table 2 and Table 3, respectively.

In addition, the iPad-version of the NIH-Toolbox–Cognitive Battery (NIHTB-CB, https://www.nihtoolbox.org/, accessed on 20 September 2023) has been used for a neuropsychological assessment. The NIHTB-CB is a comprehensive neuropsychological battery, specifically developed for outcome assessment in clinical trials and has already been validated for individuals with ID and with FX (mental age > 3 years). Although its employment is mandatory for clinical trials [22], at present this battery is available only in English and Spanish. For these reasons, non-verbal tasks for visual attention and executive function assessment only were administered. The two tasks selected were NIH-TB Flanker Inhibitory Control and Attention Test [23] to assess the attention and executive functions, and the NIH-TB Dimensional Change Card Sort Test [24] to assess executive functions and, specifically, the shifting ability (SI).

For both the NIH-TB tasks we recorded accuracy (ACC) and reaction times (RTs) for each trial (dependent variables (DVs)). For the data analysis, the initial trials of the training part of the task were removed. Binomial tests were carried out for ACC and T-tests for independent samples for RTs. As for accuracy, a two-tailed binomial test analysis revealed a significant difference in accuracy between chance and chance. In the NIH-TB Flanker Inhibitory Control and Attention task, the correct responses for Proband 1 and Proband 2 were 26 out of 30 (*p* < 0.001) and 30 out of 30 (*p* < 0.001), respectively. Instead, in the NIH-TB Dimensional Change Card Sort task, the correct responses for Proband 1 and Proband 2 were 20 out of 30 (*p* = 0.099) and 29 out of 30 (*p* < 0.001), respectively. As for Reaction Time (RT), in the NIH-TB Flanker Inhibitory Control and Attention task, a two-tailed independent samples *t*-test analysis showed a significant difference in RTs, specifically Proband 1 was faster than Proband 2 (*M*_Proband 2_ = 5.50 ± 3.06 s.; *M*_Proband 1_ = 1.01 ± 0.16; *t*(58) = 8.02, *p* < 0.01). Whereas, in the NIH-TB Dimensional Change Card Sort task, a two-tailed independent samples *t*-test analysis did not show a significant difference in RTs; specifically, Proband 1 was not faster than Proband 2 (*M*_Proband 1_ = 0.89 ± 0.95 s.; *M*_Proband 2_ = 0.83 ± 0.44; *t*(58) = 0.30, *p* = *ns*).

### 2.3. Time Perception

Time perception refers to the ability to perceive and use time and it is a fundamental dimension of everyday life that children experience [25]. It has been suggested that disorders in timing and/or time perception may be a key characteristic, or cause of, some of the behavioral and cognitive impairments in NDDs (i.e., ASD) [26]. Timing literature distinguishes between explicit and implicit timing processes [27]. As for the explicit timing tasks, participants knew in advance that they had to estimate time. Conversely, implicit timing occurs as result of non-temporal task goals. For example, when motor responses follow a strict temporal structure. For instance, task instructions may ask participants to make a perceptual judgment about a particular motor action. Although no explicit duration estimates of the stimulus or action are required, the temporal structure inherent in the motor execution will automatically engage timing mechanisms. Here, one explicit timing task (time discrimination task) and three implicit timing tasks (a foreperiod task and two rhythmic tasks—one visual and one auditory) were included (see SI).

For the time discrimination task, performance was analyzed in terms of temporal intervals correctly discriminated. Proband 1 and Proband 2 accurately discriminated all trials. Specifically, Proband 1 obtained 75% of accuracy and Proband 2 obtained 85% of accuracy. As for the foreperiod task, performance was analyzed in terms of RTs. Both Probands demonstrated the foreperiod effect (shorter RTs as the duration of the foreperiod intervals increased). Proband 1 was slower than Proband 2, but both reduced their RTs as the foreperiod intervals increased. Also, for the rhythmic visual and auditory tasks, performance was analyzed in terms of RTs for the regular and irregular conditions. In general, Probands are faster in the regular compared to the irregular conditions. In the visual rhythmic task, Proband 1 showed faster RT in the regular (488.56 ms) compared to the irregular (515.87 ms) conditions. This facilitatory effect was not observed in Proband 2 (regular condition = 578.44 ms; irregular condition = 500.60 ms). Whereas, in the auditory rhythmic task, Proband 1 (regular condition = 170 ms; irregular condition = 238.62 ms) and Proband 2 (regular condition = 242.37 ms; irregular condition = 256.78 ms) demonstrated the facilitatory effect in the regular compared to the irregular conditions.

Overall, data suggested that the ability to discriminate time durations (explicit ability to manage time) is preserved even with a lower AR; importantly the implicit processing of time was also preserved in particular when tested with the foreperiod task, indicating the ability to extract temporal information to predict the target appearance. When tested with the rhythmic task in the auditory condition, both Probands demonstrated shorter RTs with regular compared to irregular rhythms.

### 2.4. Numerical and Arithmetical Abilities

Numerical abilities are complex cognitive skills essential for dealing with requirements of the modern world. Mathematical learning difficulties have previously been described in FXS, with reported difficulties in basic numerical comprehension and magnitude estimation in female carriers of both full mutations (e.g., [28,29]) and premutations [30]. Because low mathematical achievement negatively impacts individuals’ school attainment, mental health, and even self-esteem [31,32], it constitutes a relevant aspect to consider both in clinical and research contexts [33]. In the current work, participants completed a series of computerized and standardized tasks. The computerized tasks focused on number comprehension abilities of non-symbolic representations, assessing fundamental skills such as quantity discrimination, ordering, and estimation. They were originally developed for typically developing kindergarten children and were administered via tablet. Symbolic processing was instead evaluated through a combination of experimental and standardized paper-and-pencil tasks (Battery for Developmental Dyscalculia, BDE-2) [34] that assessed counting principles as well as mental arithmetic. Together, these tasks aimed to provide an assessment of participants’ numerical processing abilities across different formats and skill domains (see SI).

- Number comprehension

Proband 1 demonstrated poor understanding of most tasks involving fundamental number comprehension. As a result, no data were available for meaningful comparison. The only two exceptions were the Ordering Sets and Ordering Numerals tasks. For the Ordering Sets, she achieved 80% accuracy on the ascending trials; however, she was unable to complete the descending trials. She also completed the ascending trials for Ordering Numerals tasks with 73.3% accuracy (see Table 4).

In contrast, Proband 2 demonstrated consistent performance across all administered tasks. She exhibited strong proficiency in quantity discrimination, correctly identifying larger dot groups (94.44% accuracy). Additionally, she completed all ordering tasks, regardless of the type of stimuli presented or the order format (ascending or descending), with 80% accuracy in Ordering objects by Size, and 100% accuracy in Ordering Sets and Ordering Arabic numerals.

- Counting principles

Proband 1 was unable to initiate the Counting Objects and the Enumeration task whilst Proband 2 exhibited adequate proficiency in both tasks, with 100% accuracy in counting and a Z score of 0.7 in the standardized enumeration task. Additionally, Proband 1 showed uneven proficiency in number recognition and quantity mapping in the “Give-me-a-number” task, achieving 100% accuracy when Arabic numbers were displayed in a visual format and 14.3% accuracy when numbers were presented verbally. Proband 2 appeared to have acquired cardinality principles; however, her performance dropped below 50% when numbers were presented verbally.

- Arithmetic Skills

More advanced numerical skills, such as mental calculations in either non-symbolic (sum of sets of dots) or symbolic (mental calculation) formats, could not be assessed in Proband 1 due to her inability to engage with these tasks. Proband 2 achieved an accuracy rate of 91.66% in non-symbolic addition tasks, indicating a good grasp of foundational arithmetic operations. This proficiency was confirmed by her performance in the standardized mental calculation task in which Z scores ranged from 0.57 to 1.02. This suggests an adequate understanding of symbolic numerals and an ability to accurately perform operations on them.

Overall, the results showed clear differences in the numerical abilities of the two Probands, reflecting variations in cognitive functioning and genetic expression.

### 2.5. Gait Analysis

In children with FXS, musculoskeletal manifestations such as flexible flat feet, joint laxity and hypotonia can lead to non-physiological gait patterns. Indeed, in these children, gait analysis has documented significant alterations, such as a characteristic pattern of excessively flexed hip and ankle joints with reduced knee flexion, suggesting overall immature motor control [35]. Furthermore, the possibility to classify children with FXS from typically developing peers by means of features extracted from joint kinematic analysis and surface electromyography (sEMG) during gait has been documented [36]. In line with these studies, both Proband 1 and 2 underwent video-based (4 GoPro Hero 7 cameras, 60 Hz) markerless gait analysis combined with sEMG analysis (FreeEmg, BTS, 1000 Hz) as described in [35]. From video sequences, lower limb joints kinematics and space time parameters were extracted, as well as the sEMG signal processed as in [35]. Gait analysis data of Proband 1 and Proband 2 were compared with normative bands derived from previously published data by the authors [35] (Figure S1).

In terms of space time parameters, Proband 1 differed from Proband 2. The first one reported value closer to those of healthy control subjects (CS) (see Table 5) except for cadence that showed higher values. Differently from Proband 1, in Proband 2, longer Stride Length and higher Velocity were detected in comparison with CS; however, both showed an increased cadence (see Table 5).

**Figure 1:**
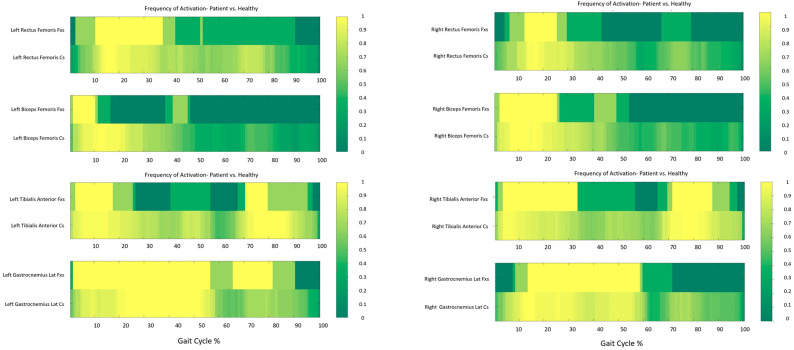
Frequency Maps describing the sEMG analysis results of Proband 1 in comparison with CS. Each graph reports in the first line the data of Proband 1 and in the second line CS data. On the left column muscles of the left side are reported: Rectus and Biceps Femoris on the top and Tibialis and Gastrocnemius Lateralis on the bottom; on the right column muscles of the right side are reported (Rectus and Biceps Femoris on the top and Tibialis and Gastrocnemius Lateralis on the bottom). On the x axis the gait cycle from 0 to 100%. Yellow = muscle activity detected for all trials, dark green = muscle activity never detected, light green = muscle activity detected for some trials.

Overall, the sEMG activity recorded larger differences in Proband 2 than Proband 1 with respect to CS. Specifically, in Proband 1, a delayed activity on the Right Rectus Femoris was detected bilaterally during initial contact and loading response, along with an inactivity during the mid-swing phase. The Biceps Femoris exhibited a similar activity to CS bilaterally, although a shorter duration was detected on the left side. Regarding the calf muscles, Tibialis Anterior exhibited a slightly delayed activity at initial contact and loading response, while during the mid-swing phase a similar activity to CS was detected. On the right limb, Gastrocnemius Lateralis displayed an activity in line with CS, while on the left side, a prolonged activity was detected (Figure 1).

**Figure 2:**
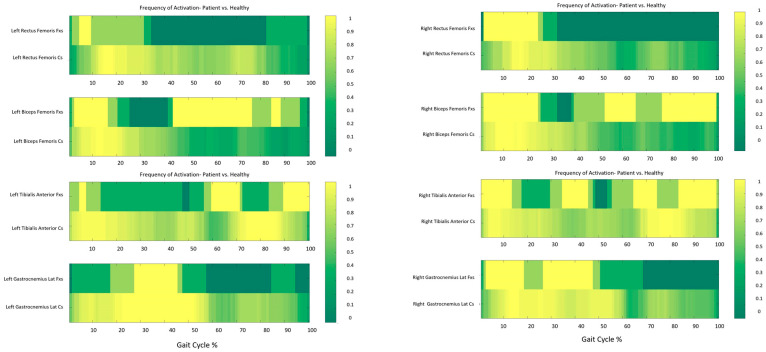
Frequency Maps describing the sEMG analysis results of Proband 2 in comparison with CS. Each graph reports in the first line the data of Proband 2 and in the second line CS data. On the left column the muscles of the left side are reported: Rectus and Biceps Femoris on the top and Tibialis and Gastrocnemius Lateralis on the bottom; on the right column the muscles of the right side are reported (Rectus and Biceps Femoris on the top and Tibialis and Gastrocnemius Lateralis on the bottom). On the x axis the gait cycle from 0 to 100%. Yellow = muscle activity detected for all trials, dark green = muscle activity never detected, light green = muscle activity detected for some trials.

Proband 2 exhibited earlier and shorter activity on the Rectus Femoris bilaterally at initial contact and loading response, as well as no activity was revealed during the swing phase of gait. The Biceps Femoris showed an activity in line with CS, apart for the swing phase, where the muscle was still active differently than CS. Regarding the calf muscles, a similar activity to CS was detected on the Left Gastrocnemius Lateralis, while on the Right Gastrocnemius Lateralis an earlier and fragmented activity was noted. The Right Tibialis Anterior exhibited an activity pattern in line with CS at initial contact and loading response, with an additional activity recorded during mid-stance; a fragmented activity was noted during the mid and terminal swing phases. On the left side, the Tibialis Anterior showed shortened and delayed activity at both initial contact and loading response, in association with a fragmented activity during mid and terminal swing (Figure 2).

### 2.6. Responding Joint Attention (RJA)

In NDDs, responding to joint attention (RJA), which refers to the ability to follow another person’s gaze or gestures to share focus on an object or event, is often impaired. Individuals with FXS tend to avoid eye contact, focusing on other parts of the face, likely to reduce anxiety during social interactions [37,38]. Anxiety, prevalent in FXS, worsens RJA by leading to social avoidance and hypervigilance, even more than in children with ASD [39]. The goal of this study was to obtain a closer look at the RJA individual differences in children with FXS using a RJA task with webcam-based remote eye-tracking. The RJA study employed a comprehensive 3 (Phases: 1, 2, and –3) × 3 (Areas of Interest, AOIs: face, cued object, and uncued object) × 5 (Delays: 200, 400, 600, 800, 1000 ms) within-participants design, with full procedure and stimuli details available online through (https://www.labvanced.com/player.html?id=43162 accessed on 20 September 2023). The design involved two blocks of ten trials each, summing up to twenty trials. Each trial presented the face of one of two female actresses, who randomly oriented towards a pinwheel on either side, against a consistent black background with elements centered for uniform proportions across different screens (see Supplementary Information).

**Figure 3:**
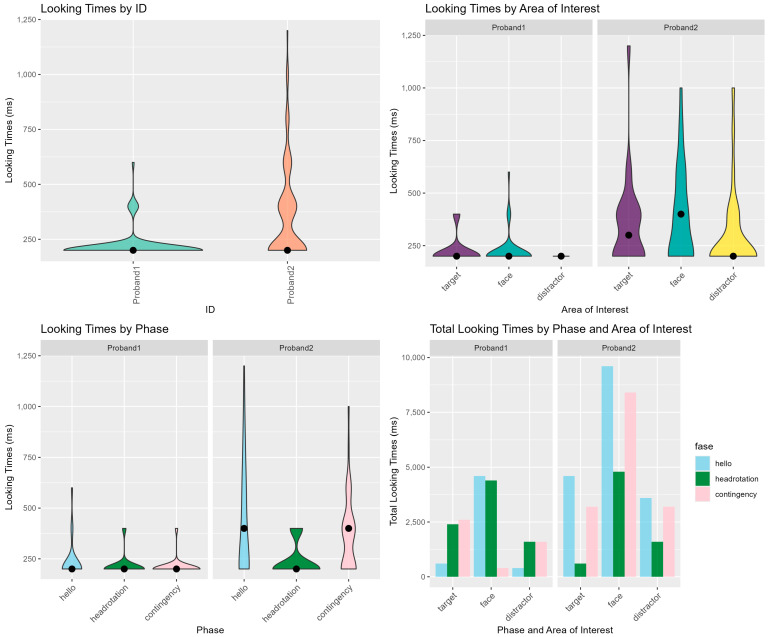
Looking times distribution across Participant, AOI, and phase.

**Figure 4:**
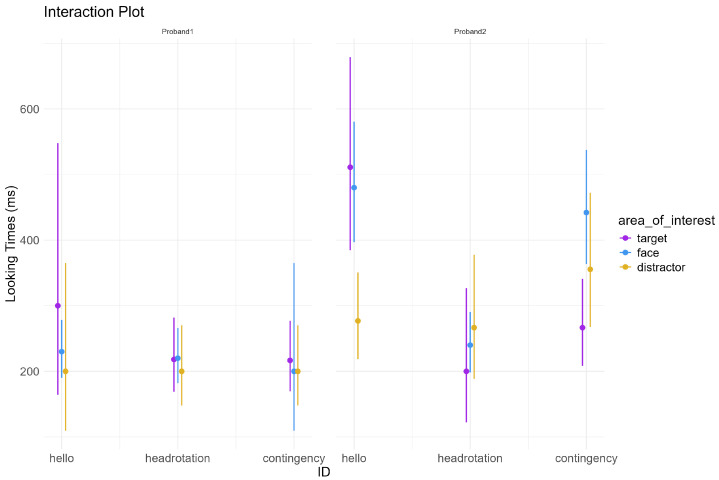
Looking times estimated effects and 95% CI predicted by the interaction between Participant, AOI, and phase. Purple color refers to ‘target’, blue color refers to ‘face’ and ocher (dark yellow) refers ‘distractor’.

Figure 3 shows the looking distribution across participants, AOI and phase. Figure 4 shows the model estimated effects of AOI, phase, and participants, as well as their interactions, on the looking times data. The main effects of AOI, phase, and participant were not statistically significant. The interaction effects between AOI and PHASE were also not statistically significant. The overall model fit was adequate.

These findings indicate that, within the limits of the data and model assumptions, looking times do not show strong significant differences across the experimental conditions and participants suggesting that other factors might need to be explored to explain the observed variability shown in Figure 3 in looking times between participants.

## 3. Discussion

Among different molecular parameters, it has been suggested that AR plays an important role in affecting cognitive functioning in FXS FM females. Despite its importance, only few studies have investigated the role of AR in females carrying the FM and, to the best of our knowledge, all studies have used traditional methods to investigate the cognitive profile, such as standardized cognitive and neuropsychological tests. Furthermore, whether AR correlates with general or specific aspects of cognitive function remains controversial. The aim of the present study was to investigate the impact of AR on phenotypical functioning of two FXS girls with seemingly identical *FMR1* genotypic profile of FM but different AR levels (70% vs. 30%). To achieve this goal, we proposed an innovative multi-method protocol that combines molecular investigation, gait analysis, lab-based experimental tasks, neuropsychological testing, and parent-reports questionnaire.

Data reported here showed that lower AR corresponds to worse performances in most, but not all the areas considered. From a cognitive and adaptive level, the two Probands differ in terms of IQ (mild intellectual disability in the former and normal IQ in the latter) and adaptive level. At the behavioral level, no differences were observed, except for borderline scores for social withdrawal problems and depression in Proband 1.

As for time perception, the data showed that both implicit and explicit time processing could be preserved even with lower AR. Interestingly, our data indicate that in a visual rhythmic task, the performance (in terms of RTs) of Proband 1 is even better than that of Proband 2. This result, which needs to be investigated in more detail, seems to indicate that some basic skills can be preserved even with a lower AR.

Furthermore, mathematical skills were found to be better preserved with higher AR. This finding is particularly relevant, as mathematics may be an area of relative weakness for the female with the premutation as well as the FM [40]. Instead, our data suggest that, at least for the tasks we administered, mathematical abilities may be preserved in females with FM but high AR. This aspect should be further investigated in future studies.

In addition, while Proband 1 and Proband 2 appear to respond similarly during the RJA task, the descriptive visualizations highlight differences in data distribution between the two, suggesting that distinct attention strategies may underlie their responses. This implies that the ability to attend to the object in response to another person’s gaze shift could still be preserved through these different strategies. Future research should investigate whether data distribution analysis reveals individual differences, particularly in more complex abilities like initiating joint attention (IJA).

Finally, gait analysis was able to detect the presence of differences both between the two Probands as well as between Probands and controls. In contrast with most of the results, the subject with lower AR displayed milder differences when compared with healthy controls. Gait analysis illustrates the complex, and precise result of human motor control, it depends on the ability to control, coordinate, and integrate the many resources required to manage the interplay between the individual and the environment. As a result of this complexity, individuals may adopt many different strategies to perform the same task and this interindividual variety is exacerbated when subjects are required to cope with possible motor impairments [41]. With this in mind, future studies are needed in order to investigate whether this result reflects the ability in subjects with higher AR to develop a different motor control strategy to cope with the orthopedic impairments associated with FXS (i.e., ligamentous laxity, hypotonia).

## 4. Conclusions

Taken together, the data suggest that AR can be associated with some, but not all aspects of cognitive functioning, while seems to be associated with the motor control of gait. However, the contribution of domain specific and domain general cognitive factors on the emergence of these deficits constitutes a fertile area of future research. Of course, the present study, especially with only two cases, does not pretend to give definitive answers about the role of AR in the clinical manifestations of FXS. However, we believe that it could be a first step towards a greater awareness of the relevance of this molecular parameter (beyond CGG repeats or methylation status) in characterizing the female FXS phenotype, not only in PM but also in FM conditions.

Importantly, as data continue to be collected, this will allow a more in-depth investigation of phenotypic features with a larger sample of FM participants and of the role of genetic and epigenetic factors over the specific mechanisms and the associated cognitive impairments. One aspect to be considered could be the quantification of FMRP protein, which plays a key role in FXS-associated cellular functions. Integrating direct measurements of the protein with AR analysis could offer a deeper understanding of the molecular interactions that influence clinical phenotypes.

Overall, the current study supports the idea that X inactivation contributes to clinical variability in females [5,6] and reinforces the importance of AR as a relevant clinical parameter in FM females that needs to be considered in molecular investigations.

## Figures and Tables

**Table 1:** Standard scores on WISC-IV and z scores on some neuropsychological tasks (Bells test, some subtest of the NEPSY-I and II batteries, the Spatial Recall test).

	Proband 1	Proband 2
Age (y,m)	10.1	11.8
Age at Diagnosis (y)	4	7
Cognitive Profile (WISC-IV)	Standard Score	
Full Scale IQ	55	110
Verbal Comprehension	70	94
Perceptual Reasoning	67	108
Working Memory	55	112
Processing Speed	72	123
Block Design	5	13
Similarities	8	9
Digit Span	4	12
Picture Concepts	5	11
Coding	5	15
Vocabulary	3	9
Letter-number sequencing	1	12
Matrix reasoning	5	10
Comprehension	4	9
Symbol Search	5	13
Neuropsychological Profile	z score	
Selective Visual Attention (Bells Test)	−0.92	−0.44
Sustained Visual Attention (Bells Test)	−2.21 **	1.69
Auditory Attention (Nepsy-II)	n/v	0.57
Response Set (Nepsy-II)	n/v	0.91
Visuospatial Memory (Spatial Recall Test)	−3.29 **	0
Visuospatial Memory-Deferred (Spatial Recall Test)	−3.97 **	−1.32 *

** Scores above the pathological threshold; * borderline or “at risk” scores. n/v: not valuable.

**Table 2:** T scores on Child Behavioral Checklist (CBCL 6–18) and Conners’ parent rating scales (CPRS-R: L).

	Proband 1	Proband 2
CBCL 6–18	T scores	
Syndromic Scales		
Anxious-depressed	54	50
Retired-depressed	66 *	50
Somatic complaints	50	50
Social problems	57	50
Thought Problems	58	50
Attention problems	52	50
Rule breaking behavior	52	50
Aggressive behavior	50	50
Internalizing	58	33
Externalizing	47	34
Total problems	51	25
DSM-Oriented Scales		
Affective	52	50
Anxiety	59	50
Somatic complaints	50	50
ADHD	50	50
Oppositional defiant	52	50
Conduct problems	50	50
CPRS-R:L		
ADHD scale	48	40
Attention Problems	47	40
Hyperactivity/impulsivity Scale	41	38

* borderline or “at risk” scores.

**Table 3:** Standard scores of adaptive functioning measured by VABS-II.

	Proband 1	Proband 2
VABS-II	Standard Score	
Communication	74	103
Expressive Language	10	17
Receptive Language	8	14
Written	13	15
Daily life skills	60	95
Personal	7	13
Domestic	7	16
Community	8	13
Socialization	68	112
Interpersonal relationships	8	17
Play and leisure time	8	16
Coping skills	11	16
Global Composite Scores	66	104
Level of adaptive functioning	Low	Adequate

**Table 4:** Numerical and arithmetical tasks. Performance is reported as raw scores and percentages for computerized tasks, and as z-scores for standardized tasks.

Process Assessed	Tasks	Accuracy	
		Proband 1	Proband 2
Number comprehension	Compare sets of dots	(0/18) n/v	(17/18) 94.44%
	Ordering by Size-Ascending	(0/30) n/v	(24/30) 80%
	Ordering by Size-Descending	(0/30) n/v	(24/30) 80%
	Ordering Sets-Ascending	(24/30) 80%	(30/30) 100%
	Ordering Sets-Descending	(0/30) n/v	(30/30) 100%
	Ordering Numerals-Ascending	(22/30) 73.33%	(30/30) 100%
	Ordering Numerals-Descending	(0/30) n/v	(30/30) 100%
Counting principles	Counting-Dice-Configuration	n/v	100%
	Counting-No-Configuration	n/v	100%
	Enumeration ^(^*^)^	n/v	Z score = 0.7
	Give-me a Number-Visual-Arabic Format	(7-CPK) 100%	(7-CPK) 100%
	Verbal Format	(1-CPK) 14.29%	(6-CPK) 42.86%
Arithmetic skills	Non-symbolic Addition Task	(0/12) n/v	(11/12) 91.66%
	Mental Multiplication ^(^*^)^	n/v	(17/18) Z score = 0.57
	Mental Calculations ^(^*^)^	n/v	(16/18) Z score = 1.02

Note: the symbol ^(^*^)^ refers to standardized tasks from the BDE-2 [34]; CPK = Cardinal Principle Knowers. n/v = not valuable.

**Table 5:** Space-time parameters in gait analyses for Proband 1 and 2 compared to control subjects.

	Stride Length (m)	Stride Time (s)	Velocity (m/s)	Stance (%)	Swing (%)	Cadence (Step/min)
Proband 1	1.13 ± 0.05	1.07 ± 0.04	1.05 ± 0.04	65.44 ± 3.5	34.56 ± 3.5	56.17 ± 2.1
Proband 2	1.46 ± 0.06	0.99 ± 0.02	1.48 ± 0.08	62.74 ± 1.5	37.26 ± 1.5	60.87 ± 1.23
Control (CS)	1.2 ± 0.1	1.1 ± 0.1	0.99 ± 0.1	61.6 ± 1.4	38.4 ± 0.1	49.7 ± 4.7

## Data Availability

The original contributions presented in this study are included in the article/Supplementary Material. Further inquiries can be directed to the corresponding author.

## Supplementary Materials

The following supporting information can be downloaded at: https://www.mdpi.com/article/10.3390/ijms26020771/s1, Figure S1: Acquisition and data processing pipeline. References [34,35,40,41,42,43,44,45,46,47,48,49,50,51,52,53,54] are cited in the supplementary materials.
