# Supplementary material for: Neurobehavioral Outcomes Relate to Activation Ratio in Female Carriers of Fragile X Syndrome Full Mutation: Two Pediatric Case Studies
Source: Int J Mol Sci. 2025 Jan 17;26(2):771. doi: 10.3390/ijms26020771 (PMC11766333; doi:10.3390/ijms26020771)
Supplement: Supplementary file 1 [file ijms-26-00771-s001.zip › ijms-3412626-supplementary.pdf]

**Title: “Neurobehavioral outcomes relate to activation ratio in female carriers of Fragile X Syndrome Full mutation: Two pediatric case studies” by E. Di Giorgio et al.,**

## Supplementary Information

The present study is part of a broader research aimed at characterizing the functional profiles of pediatric individuals with FM (with or without mosaicism) and PM, using a multi-method protocol to better understand the genotype-phenotype link. This section describes the procedures and methods used to collect data in each area under investigation.

## 1. Molecular analyses

Genomic DNA (gDNA) was extracted from peripheral blood leukocytes (PBL) and flaking cells of the oral mucosa on an automated Maxwell ® 16 Blood DNA Purification System (Promega, Milan, Italy) and quantified by spectrophotometer with NanoDrop TM (ThermoFisher Scientific, Waltham, MA, USA). To identify the full range of *FMR1* CGG repeat expansions and the presence of AGG interruptions, gDNA (40–60 ng) was amplified with Amplidex *FMR1* PCR kit (Asuragen, Austin, TX, USA) according to the manufacturer’s recommended protocol. All amplicons were analysed by capillary electrophoresis (CE) on a 3130xl Genetic Analyzer (Applied Biosystems, ThermoFisher Scientific, Waltham, MA, USA) GeneMapper® v 4.0 software with ROX 1000 size ladder (Asuragen, Austin, TX, USA) was used for automated sizing of *FMR1* gene-specific peaks in conjunction with mobility correction factors [42].

Samples were classified as follows: <45 CGG repeats, normal; 45 to 55 CGG repeats, intermediate range; 55 to 200 CGG repeats, PM; and >200 CGG repeats, FM according to American College of Medical Genetics guidelines [43]. DNA samples were analyzed for methylation status using the AmplideX *FMR1* mPCR reagents (Asuragen) according to the manufacturer’s recommended protocol. Briefly, 160 ng DNA samples were premixed with two plasmids: a digestion control (DigCtrl) and PCR reference control (RefCtrl). This premix was separately aliquoted to a control or methylation-sensitive digestion reaction. Capillary electrophoresis (CE) on a 3130xl Genetic Analyzer (Applied Biosystems, ThermoFisher Scientific, Waltham, MA, USA) were performed. All alleles were detected using FAM-labeled primers, but only the proportion of the protected methylated allele was available for PCR using HEX-labeled primers. Lack of methylation at either HpaII site resulted in digestion and thus no amplification.

The percent methylation (%Me) for each peak$_i$ was calculated as a ratio of peak heights between digested (HEX) and undigested samples (FAM), normalized to the CGG control amplicon peak height according to Eq.1:

where $Peak_{i,HEX}$ is the signal height in the HEX channel corresponding to the methylated fraction of $Peak_{i,FAM}$ from the control digestion reaction and REF HEX and REF FAM to the peak heights of the PCR reference peak in the HEX and FAM channels, respectively.
Alleles are reported as unmethylated (<10%), partially methylated (10–80%) and fully methylated (>80%).

## 2. Neuropsychological Assessment with NIH-CBT

*Flanker Inhibitory Control and Attention Test.* The task comprised 40 trials, with an average completion time of 4 minutes. In each trial, a central directional target, represented by a fish overlaid with an arrow, was flanked by similar stimuli (irrelevant targets) on either side. On congruent trials, the irrelevant targets faced the same direction as the central target, while on incongruent trials, they faced the opposite direction. Participants were instructed to indicate the direction of the central fish target.

*Dimensional Change Card Sort Test.* A target visual stimulus (either a ball or a truck, coloured orange or blue) is to be matched with one of two alternative stimuli, according to either shape or colour. The experiment commenced with a series of trials in which only one dimension was relevant, followed by a second block in which the other dimension was critical. After the switch blocks, the task continued with a mixed block, in which colour was the primary criterion for most trials with unpredictable shifts to shape. The relevant criterion word, either "color" or "shape," was displayed on the screen and delivered orally. A total of 40 trials were administered.

## 3. Time perception

*Time discrimination task*. Participants were asked to discriminate the duration of two consecutive intervals. The first stimulus lasted 1000 ms and the comparison intervals lasted 400, 700, 1300 and 1600 ms. Participants used two keys on the keyboard masked with “B” = short and “L” = long to indicate that the second interval was shorter or longer than the first stimulus presented. The stimuli were an image of a star (first stimulus) and an image of a sun (second stimulus). The task included 8 repetitions for each comparison interval for a total of 32 trials. A practice phase was included before the testing phase including 1 repetition for each temporal interval.

*Foreperiod task.* Each trial started with the presentation of a photo camera that lasted 400, 700, 1000, 1300 or 1600 ms followed by the presentation of an image of a lion at the centre of the photo camera’s lens. Participants were instructed to take a photo of the lion and press the spacebar as soon as the image appeared. Following the response to the target, or after 2000 ms in case of a missed response, we presented a variable ITI of 500 ms and the next trial began. The foreperiod task

comprised three blocks of variable foreperiod in which all durations were randomly presented 2 times for a total of 30 trials. An initial training phase with 5 trials was used to ensure that participants correctly understood the task.

*Visual and auditory rhythmic tasks.* Participants performed the rhythmic tasks with visual or auditory stimuli. In *the visual rhythmic task*, a fixation cross was followed by six blank screens interleaved with five stimuli representing a rabbit' paw each presented for 100 ms. This alternation of blank screens and visual stimuli created a flickering effect that could be regular or irregular. In the regular sequence, each blank screen had a fixed duration of 500 ms. Conversely, in the irregular sequence, the duration of the blank screens varied randomly across different values (100, 300, 500, 700, or 900 ms). The last screen preceding the target (an image of a rabbit) had a fixed duration of 1000 ms for both regular and irregular sequences. In the *auditory rhythmic block*, five sounds were transmitted through headphones, each lasting for 100 ms, and interleaved with silent periods spaced either regularly or irregularly. During the sound presentation, a blank screen was displayed. Following the auditory rhythms, the target (an image of a rabbit) appeared. Participants were instructed to detect the target, being informed that the presentation of the preceding rhythmic sequence was task irrelevant. The visual and auditory rhythmic tasks included 9 trials with regular and 9 trials with irregular intervals for each task. An initial training phase was included before each task with 2 trials for regular and irregular conditions.

## 4. Numerical and arithmetical abilities

*Number comprehension.* Numerical knowledge was evaluated using tasks designed to assess quantity discrimination. These tasks required participants either to compare two sets of objects or to arrange multiple sets in a specified order.

A classic non-symbolic comparison task measures numerical acuity by asking participants to compare two sets of dots [44]. In each trial, 2 sets of dots (one on the left and one on the right) were simultaneously presented for 2500 ms. Each of the sets contained from 2 to 64 dots. Participants were requested to identify the larger one. The comparison subtended various ratios (e.g., 1:2, 2:3) to evaluate relative quantity discrimination, thus approximate quantities without counting. Participants completed 18 trials with no time constraints.

Ordinality knowledge was assessed through three distinct tasks, designed to evaluate basic sequencing skills:

a) Ordering by Size. This digital task assesses the ability to arrange objects based on their relative size. Participants completed 12 trials—six requiring ordering from smallest to largest (ascending) and

six from largest to smallest (descending). There were two trials in which participants were given 3 identical objects to order, two trials with 4 objects, and two trials containing 5 objects.

b) Ordering Sets: This digital task evaluates participants' ability to order quantities displayed in a non-symbolic format (dots). Participants are presented with 5 sets of dots, each containing quantities ranging from 1 to 12. They were instructed to arrange them in either ascending or descending order. The task included 12 trials (six ascending and six descending). The first two sets ranged from 1 to 5; the following two trials contained sets from 6 to 10; and the last two trials contained random sets chosen within the range 1 to 10.

c) Ordering Numerals. This digital task measures participants' semantic understanding of numerical symbols and their sequencing. Participants are presented with five Arabic numerals (out of ten possible ones ranging from 1 to 10) and asked to arrange them in ascending (smallest to largest) or descending (largest to smallest) order. Each participant completed six trials in each order. The first trials included numbers from 1 to 5; the following two trials contained numbers from 6 to 10; and the last two trials contained random numbers chosen with the range 1 to 10.

*4.1 Counting Principles*

Counting skills and principles were assessed by means of the following tasks:

a) Counting Objects Task. It evaluates participants' ability to accurately count objects presented in different spatial configurations. Across 20 trials, participants are shown a series of objects on a screen. Half of the trials feature structured arrangements (e.g., dice-like configurations), while the other half feature unstructured arrangements.

b) Enumeration (BDE-2) [34]. This standardized task assesses individuals' verbal knowledge of numerical sequences, testing the automaticity and stability of both forward and backward counting. The task initially requires participants to verbally recite the number sequence from 1 to 40 (or from 80 to 140 for older children), with the time taken being recorded. In the second phase, participants are asked to reproduce the number sequence backward, starting from 40 (or 140), and are stopped based on the time they registered in the first phase. Accuracy is determined by the number of digits correctly pronounced in the backward sequence.

c) "Give-me a Number" Task [47]. Two versions of this task were developed to assess participants' understanding of the cardinality principle, namely that the last number in a counting sequence represents the total quantity of items in a set:

(1) Visual-Arabic format: Participants were shown Arabic numbers displayed on a basket and were asked to drag an equivalent number of apples into the basket. Numbers ranged from 1 to 7. The sequence of trials adapted to participants’ responses, providing a nuanced assessment of symbolic cardinality comprehension.

(2) Verbal format Version: The number of apples to be dragged was introduced in a verbal mode through an automated voice, requiring participants to associate spoken numerical information with corresponding quantities.

*4.2 Arithmetic skills*

Basic arithmetic skills were assessed using three tasks, separately assessing non-symbolic and symbolic operations:

a) Non-Symbolic Addition Task. Participants viewed animated sequences of dots ranging from 1 to 16, which were concealed behind an occluder before merging. After observing the sequence, participants were prompted to select the correct total from two possible options. This task, spanning 12 trials, evaluated participants' foundational understanding of additions without relying on numeric symbols.

b) Two mental calculations tasks were administered (BDE-2) [34]. Mental Multiplications required participants to complete 18 arithmetic facts (multiplication tables), each within a 3 second time limit. In the second task, the Mental Calculation Task, the participant completed 9 addition and 9 subtraction problems, each within a 30-second time limit. These tasks assessed participants' proficiency in performing basic arithmetic operations.

## 5. Gait Analysis

Markerless gait analysis was performed (4 GoPro Hero7 camera videos recorded at 30 fps) combined with surface electromyography (sEMG) analysis (FreeEmg, BTS, 1000 Hz) as described in [46]. Multiple gait trials at a self-selected speed were recorded, with at least three left and three right gait cycles included in the analysis. From video sequences, lower limb sagittal plane kinematics was computed (Track on Field software, BBSoF s.r.l.) as well as space time parameters [48,49]: hip, knee and ankle flexion extension angles, stride length [m], stride time [s], velocity [m/s], stance and swing phases duration in percentage of the gait cycle (respectively the time when the foot is in contact with the ground and the flight time), cadence [step per minutes]. The sEMG activity of Tibialis Anterior (TA), Gastrocnemius Lateralis (GL), Rectus Femoris (RF), and Biceps Femoris (BF) was acquired through 16 electrodes (24 mm in diameter and spaced 1 cm apart) applied following the guidelines reported in [50,51]. The sEMG signals were band pass filtered at 20–450 Hz with a double 5th -order Butterworth filter and full wave rectified [49]. A double-threshold statistical detector was chosen to retrieve the onset and the offset time instants of muscle activity, as it yields to better onset/offset estimation than the single-threshold algorithms [53]. An amplitude threshold $\theta$, a numerosity threshold $\rho$, and the length of the observation window m were defined. $\theta$ and $\rho$ were chosen for each signal to minimize the value of false-alarm probability and to maximize the

probability of activation detection for specific background noise. Background noise used to reveal the signal-to-noise ratio was estimated using an algorithm that follows a statistical approach which does not require any a priori knowledge of the signal [54]. Signals were digitalized setting m = 30 ms, as a suitable time-window to study muscle activation during locomotion [53, 54]. If one sample from the window exceeds the threshold, the sample was classified as a muscle contraction and set equal to 1. Signal classified as muscle relaxation was set equal to 0. A post processor applied to the digitalized signal allowed to discard the bursts shorter than 30 ms; onset instants were identified as transitions of activation from 0 to 1, offset from 1 to 0. Furthermore, digitalized signals were used to provide activation maps as in [56]. Based on the application of the double-threshold algorithm, the following parameters were extracted: activation and deactivation timing (milliseconds and gait cycle percentage) and burst duration. Fragile X individuals' gait analysis data were compared with the data of a group of healthy children (CS) matched forage and BMI as in [48].

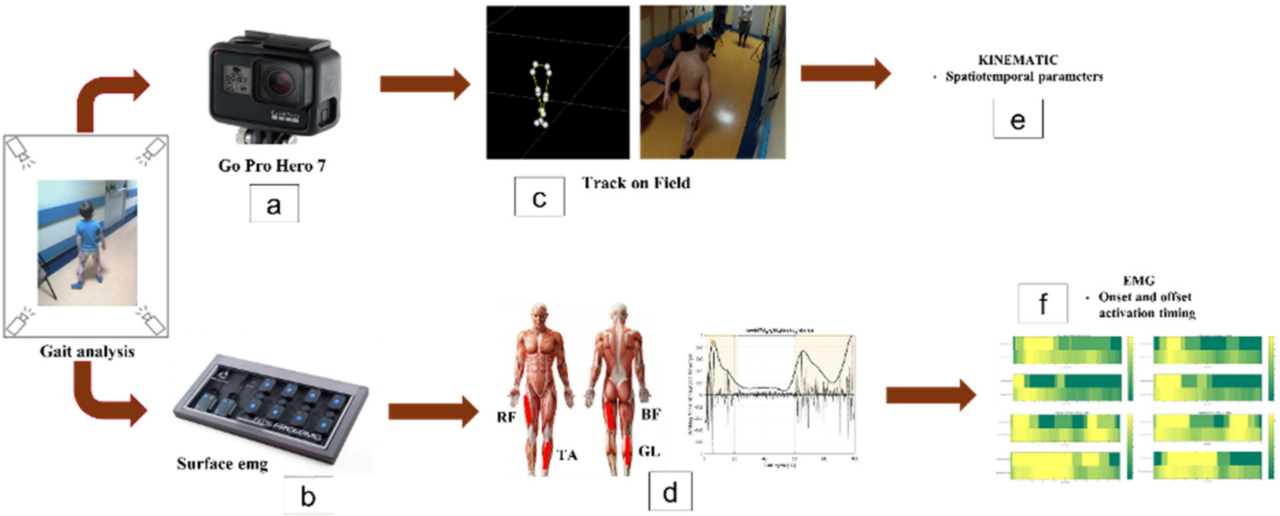


*Figure S1*. Acquisition and data processing pipeline: (a) After recording video sequences and (b) sEMG signals during gait, raw data (c,d) are processed to obtain time-space parameters (e), sEMG variables (f).

## 6. Responding Joint Attention (RJA)

The RJA study employed a comprehensive 3 (Phases: 1, 2, and 3) × 3 (Areas of Interest, AOIs: face, cued object, and uncued object) × 5 (Delays: 200, 400, 600, 800, 1000 ms) within-participants design, with full procedure and stimuli details available online through (https://www.labvanced.com/player.html?id=43162). The design involved two blocks of ten trials each, summing up to twenty trials. Each trial presented the face of one of two female actresses, who

randomly oriented towards a pinwheel on either side, against a consistent black background with elements centered for uniform proportions across different screens.

The face size was 252x154 Labvanced arbitrary units, with pinwheels at 212x137 units each, and 190 units apart from the face center. All stimuli appeared simultaneously and remained visible throughout the trial. Webcam-based eye-tracking captured fixation on the three AOIs during three phases: Phase 1 (5000ms) as the actress greeted with "Ciao! Guarda che bello!", Phase 2 (2000ms) as she turned toward a pinwheel, and Phase 3 (4000ms), maintaining gaze on the pinwheel with possible spinning initiated by the participant's successful gaze orientation.

Performance accuracy in Phase 3 was calculated as the proportion of gaze time on the target pinwheel relative to total AOI dwell time. Dwell times in Phases 1 and 2 and fixations in Phases 2 and 3, identified by low-dispersion gaze points [54], were analyzed to assess attention distribution and engagement dynamics.

The statistical analysis approach employed in the RJA study involves a combination of data preprocessing, descriptive visualization, and inferential modeling. Initially, the data are cleaned and transformed. The analysis focuses on the looking times data, which are calculated based on fixation durations associated with different AOI categories, including face, target, and distractor. The data are then summarized at the participant level using groupings of participants, phase, AOI, and items, allowing for the exploration of how looking times vary across these factors. Visual inspection is performed via violin plots that depict the distribution of looking times by participants, AOI, and phase. A bar plot is also included to show total looking times by phase and AOI, facilitating an understanding of the aggregate looking time distribution across experimental conditions. For inferential analysis, a generalized linear model (GLM) with a Gamma distribution and a log link is fitted to the data to examine the effects of AOI, phase, and their interaction on looking times. This model accounts for potential non-normality of the looking times data and allows for the assessment of the main effects and interactions among factors. Model effects are further visualized through the effects package, providing insights into the relationship between the predictors and the response variable.
